# From 2015 to 2023, eight years of empirical research on research integrity: a scoping review

**DOI:** 10.1186/s41073-025-00163-1

**Published:** 2025-04-30

**Authors:** Baptiste Vendé, Anouk Barberousse, Stéphanie Ruphy

**Affiliations:** 1 Sorbonne Université, CNRS, Sciences, Normes, Démocratie, UMR8011, Office français de l’intégrité scientifique, Paris, F-75005 France; 2 Sorbonne Université, CNRS, Sciences, Normes, Démocratie, UMR8011, Paris, F-75005 France; 3 République Des Savoirs, Ecole Normale Supérieure, Université PSL, Paris, France

**Keywords:** Research integrity, Scoping review, Meta-analysis, Responsible science, Empirical

## Abstract

**Background:**

Research on research integrity (RI) has grown exponentially over the past several decades. Although the earliest publications emerged in the 1980 s, more than half of the existing literature has been produced within the last five years. Given that the most recent comprehensive literature review is now eight years old, the present study aims to extend and update previous findings.

**Method:**

We conducted a systematic search of the Web of Science and Constellate databases for articles published between 2015 and 2023. To structure our overview and guide our inquiry, we addressed the following seven broad questions about the field:-What topics does the empirical literature on RI explore?

- What are the primary objectives of the empirical literature on RI?
- What methodologies are prevalent in the empirical literature on RI?
- What populations or organizations are studied in the empirical literature on RI?
- Where are the empirical studies on RI conducted?
- Where is the empirical literature on RI published?
- To what degree is the general literature on RI grounded in empirical research?

Additionally, we used the previous scoping review as a benchmark to identify emerging trends and shifts.

**Results:**

Our search yielded a total of 3,282 studies, of which 660 articles met our inclusion criteria. All research questions were comprehensively addressed. Notably, we observed a significant shift in methodologies: the reliance on interviews and surveys decreased from 51 to 30%, whereas the application of meta-scientific methods increased from 17 to 31%. In terms of theoretical orientation, the previously dominant “Bad Apple” hypothesis declined from 54 to 30%, while the “Wicked System” hypothesis increased from 46 to 52%. Furthermore, there has been a pronounced trend toward testing solutions, rising from 31 to 56% at the expense of merely describing the problem, which fell from 69 to 44%.

**Conclusion:**

Three gaps highlighted eight years ago by the previous scoping review remain unresolved. Research on decision makers (e.g., scientists in positions of power, policymakers, accounting for 3%), the private research sector and patents (4.7%), and the peer review system (0.3%) continues to be underexplored. Even more concerning, if current trends persist, these gaps are likely to become increasingly problematic.

**Supplementary Information:**

The online version contains supplementary material available at 10.1186/s41073-025-00163-1.

## Background

Since the 1990 sresearch integrity (RI) has emerged as a significant concern, undergone institutionalization, and evolved into a distinct field of research. The increasing number of retractions is now exceeding 10,000 per year [1]. This has sparked debates regarding whether this trend signals growing misconduct within the scientific community or reflects enhanced vigilance and improved regulatory mechanisms. For a broader discussion on these interpretations, see Oransky [2]. This debate is emblematic of the diverse issues addressed in the burgeoning RI literature, which now encompasses substantial corpora of both empirical and theoretical work. A simple search on Web of Science using the term “research integrity” returns thousands of results. We quickly realized that examining the theoretical and empirical literature would be too extensive for our study. As such, we chose to follow the footstep of the previous scoping review and focus on the empirical corpus. Young and rapidly expanding fields such as RI can greatly benefit from studies seeking to understand their evolution. Such research can identify emerging trends, pinpoint knowledge gaps, and enable researchers to adopt a more reflective stance. To that end, we structured our review around seven thematic questions:

- *Q1: What topics does the empirical literature on RI explore?* Does it focus on fabrication, falsification, and plagiarism (FFP), more subtle malpractice like questionable research practices (QRP), or something else entirely?
- *Q2: What are the primary objectives of the empirical literature on RI?* Is it looking for solutions or does it try to establish a diagnostic? Does it focus on individual transgressions or systemic flaws?
- *Q3: What methodologies are prevalent in the empirical literature on RI?* How are empirical investigations conducted, and to what extent are experimental methods used?
- *Q4: What populations or organizations are studied in the empirical literature on RI?* How much is it focused on researchers and which kinds?
- *Q5: Where are the empirical studies on RI conducted?* Which countries publish the most research on RI?
- *Q6: Where is the empirical literature on RI published?* Which journals publish the most empirical research on RI? Which disciplines contribute the most to RI literature?
- *Q7: To what degree is the general literature on RI grounded in empirical research?* What is the proportion of empirical versus theoretical contributions?

The most suitable methodology for answering broad questions about a research field is a scoping review. Munn et al. [3] describe this method as follow: “scoping reviews are an ideal tool to determine the scope or coverage of a body of literature on a given topic and […] an overview (broad or detailed) of its focus”. Unfortunately, such studies are rare in the field of RI. The most recent general systematic review, A Decade of Empirical Research on Research Integrity: What Have We (Not) Looked At? by Aubert Bonn & Pinxten [4], covers the period from 2005 to 2015. Subsequent overviews, such as those by Neoh et al. [5], Mansour & Ruphy [6], and Ali et al. [7], either focus solely on co-citation pattern analysis, lack a systematic approach, or focus on academic integrity (e.g., student cheating or RI training below the PhD level). Given the surge in publications in recent years, our study fills an important gap by examining empirical RI research from 2015 to 2023 and contrasting these findings with earlier trends.

## Methods

The methodology used in this study was designed with two objectives: to provide accurate answers to the thematic questions on RI and to maximize the validity of comparisons with the previous scoping review. To achieve these aims, we based our approach on the methodology of Aubert Bonn & Pinxten [4], making as few modifications as possible. Each modification and its rationale are detailed in tables in the Additional File 2. These tables can be used to convert the data between the two studies and estimate the validity of the comparisons. Every comparison made is valid according to the bias estimation reported in the Additional file 4.

As a scoping review, our methodology can be divided into four steps: selecting databases, automatically identifying studies through keyword searches, screening out irrelevant papers, and classifying the included studies. Both the screening and classification processes were carried out by the first author. For a more detailed description of standard scoping review methodology, see chapter 10 of the JBI Manual for Evidence Synthesis [8]. This study also adheres to the PRISMA guidelines for scoping reviews [9], with the checklist available in Additional file 1.

### Database selection

We utilized two databases: Web of Science and Constellate. Web of Science indexes over 24,000 journals, making it indispensable for systematic reviews. However, as demonstrated by Martín-Martín et al. [10], Chavarro et al. [11], and Mongeon & Paul-Hus [12], works from the humanities and social sciences are not always possible to retrieve. To address this limitation, we included Constellate, a database curated by JSTOR that specializes in the humanities and social sciences.^1^ For a discussion comparing our selected databases with those used by Aubert Bonn & Pinxten [4], see Additional file 4.

### Identification

Aubert Bonn & Pinxten [4] noted two challenges for scoping reviews in the field of RI: inconsistent definitions of RI and diverse publication formats (pp. 2). As of 2024, there have been significant improvements in defining RI. Notable resources include national integrity codes and offices, such as the OFIS (Office Français de l’Integrité Scientifique) [13] and the European Code of Conduct for Research Integrity by ALLEA (All European Academies) [14]. The latter defines RI as:

“The basic responsibility of the research community to formulate the principles of research, to define the criteria for proper research behavior, to maximize the quality, reliability, and robustness of research and its results, and to respond adequately to threats to, or violations of, good research practices.” (pp 3).

Despite these advances, it is unclear whether this definition has been uniformly adopted by the empirical RI research community. To mitigate potential biases, we incorporated a broad set of keywords from related fields that might be used in RI literature.^2^

Regarding the diversity of publication formats in the field, Aubert Bonn & Pinxten [4] identified documents classified as commentaries or notes that presented original empirical data. This means that by searching only for peer-reviewed articles we could miss includable papers. To address this, we broadened our search criteria to include any type of document allowed by the databases.^3^

**Fig. 1:**
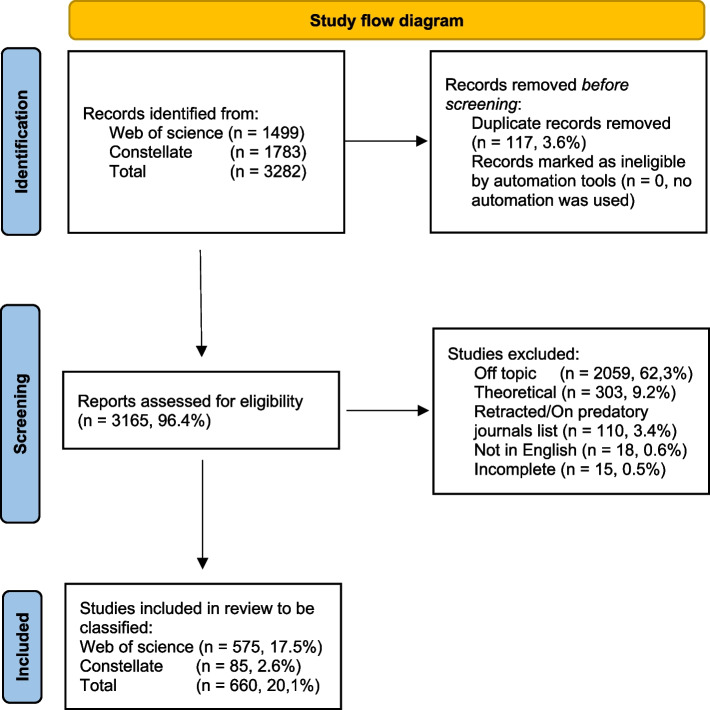
Study flow diagram. *Legend.* Study flow diagram recommended by PRISMA [15] representing the flow of information through the phases of the scoping review. List of keywords: responsible innovation, questionable research practices, research misconduct, scientific integrity, responsible research, responsible conduct of research, scientific misconduct, research integrity, scientific fraud, metascience, method, methods, result, results

The keywords were chosen with two objectives in mind: to identify RI studies and to filter for empirical research. We assumed that empirical studies would consistently include headings such as “Method” and “Result”, while theoretical studies might not. To follow Aubert Bonn and Pinxten’s methodology and to manage the volume of studies given our available resources, only English-language works were included. The search results were then automatically filtered to include studies published between 2016 and 2023 included. The exact queries used for both databases can be found in Additional File 4 (Fig. 1).

### Screening

We included every study whose research topic aligns with ALLEA’s definition of RI. This encompasses research mentioning fabrication, falsification, and plagiarism (FFP), as well as questionable research practices (QRP).^4^ It also includes FFP/QRP issues arising in the private research sector, as well as integrity training from the PhD student level. We excluded all other topics, even those closely adjacent. In particular, academic integrity (e.g., student cheating or RI training below the PhD level), field-specific methodological enhancements indirectly related to RI, and studies on research ethics (e.g., protecting the common good, addressing safety concerns, or strengthening the connection between society and science) or responsible science^5^ (e.g., combining research ethics and RI).

A second set of inclusion criteria was applied to assess the empirical nature of the studies. Articles that did not involve data collection, manipulation or that utilized non-original data for illustrative or argumentative purposes, were excluded. This last condition often led to the exclusion of news articles and editorials. However, studies that designed a theoretical tool and testing it through examples or case studies were considered to generate new data and included. Lastly we conducted a brief correspondence test based on the Predatory Journal List [16] to exclude articles from predatory journals, as we did not want to indirectly promote these types of journals. Moreover, such papers are less likely to have undergone rigorous peer review.

### Classification process

As previously mentioned, we based our classification process on the work of Aubert Bonn & Pinxten [4]. However, we implemented modifications to align the methodology with our seven thematic questions, ensure consistency with our definition of RI, or adhere to contemporary naming conventions. Details of these modifications and their justifications can be found in Additional file 2. In total, there are 10 categories and over 50 characteristics. Their detailed description is available in Additional file 4.

## Results

### Screening

From the 3,282 articles initially identified, 20% met the inclusion criteria and were incorporated into the study. The complete dataset can be found in Additional file 3. In the following sections, we present the answers to each of the seven questions. Comparisons with the previous scoping review are addressed in the Discussion section.

**Fig. 2:**
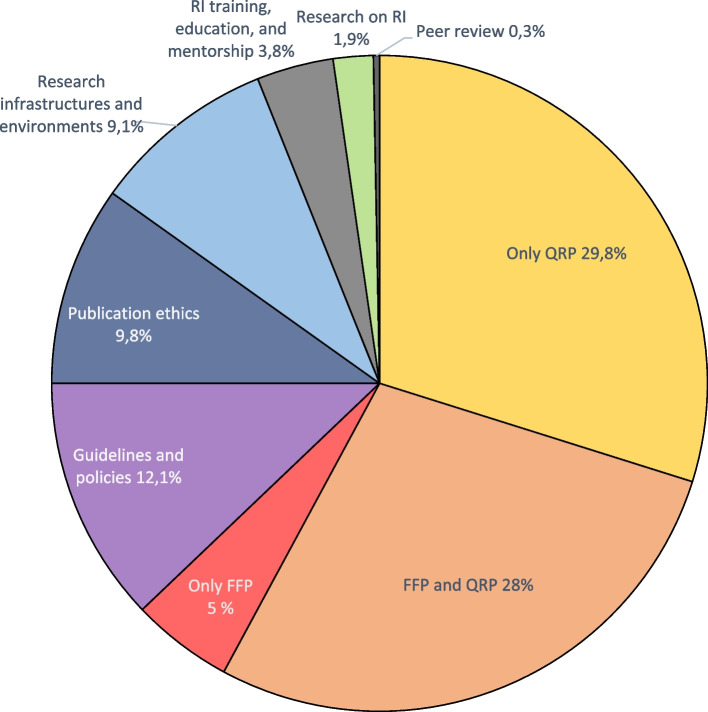
Topics of interest.* Legend.* This category gives a general overview of the themes most addressed in the field of RI. The total of articles (100%) is 660. RI = research integrity; QRP = Questionable research practices; FFP = Fabrication, falsification, plagia

- *Q1: What topics does the empirical literature on RI explore?* Does it focus on fabrication, falsification, and plagiarism (FFP), more subtle malpractice like questionable research practices (QRP), or something else entirely? (Fig. 2)

Unsurprisingly, the literature predominantly addresses the direct manifestations of the lack of RI, specifically FFP and QRP. Studies of this nature account for 62% of the total. QRP are featured in 43.8% of the literature (Only QRP 29,8% + FFP and QRP 28%/2) and are the most extensively researched topic, while FFP studies only appears in 19% of the articles (Only FFP 5% + FFP and QRP 28%/2). Another notable finding is the very low number of empirical studies on peer review, which account for only 0.3%

**Fig. 3:**
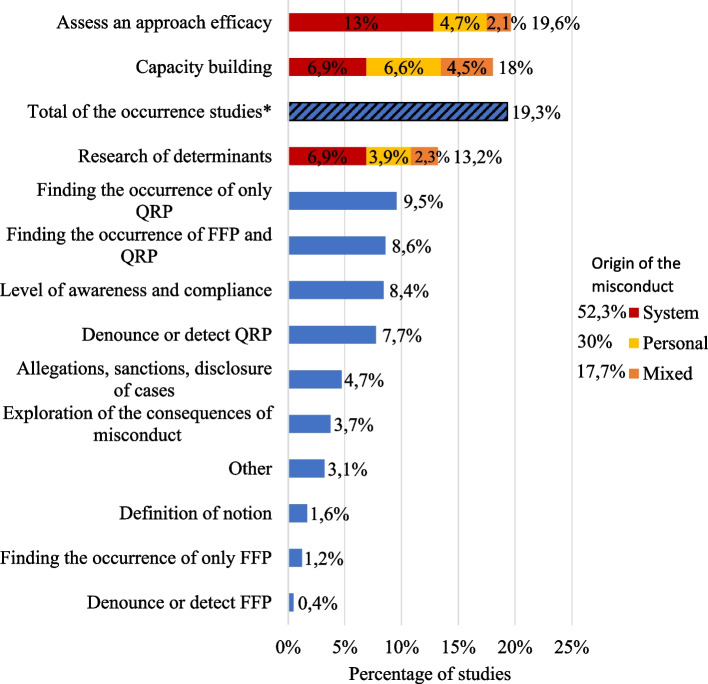
Aims and origin of the misconduct. *Legend*. This category's objective is to characterize the aim of the studies. In addition, three aim characteristics: assess an approach efficacy, research of determinants and capacity building are sub-divided into systemic factors, individual factors and mixed. The sub-division goal is to determine where does the studies place the origin of the misconduct. *The “Total of the occurrence studies” striped line is the addition of lines present elsewhere in the figure (“Occurrence of QRP”, “Occurrence of FFP” and “Occurrence of FFP and QRP”). The total of articles (100%) is 660. The total of articles for the sub-division (100%) is 335.5. RI = research integrity; QRP = Questionable research practices; FFP = Fabrication, falsification, plagia

- *Q2: What are the primary objectives of the empirical literature on RI? Does the empirical literature on RI primarily focus on identifying individual transgressions or systemic flaws?* Does it focus on individual transgressions or systemic flaws? (Fig. 3)

Most studies aim to identify the best approaches for improving research integrity (RI). More specifically, 19.6% focused on assessing the efficacy of an approach, while 18% seek to find new strategies (capacity building). Additionally, 19.3% of the studies look into the occurrence of misconduct. In terms of using a systemic or an individual framework, 52.3% of the studies focus exclusively on systems, 29.9% on individual, and 17.7% consider both perspectives.

**Fig. 4:**
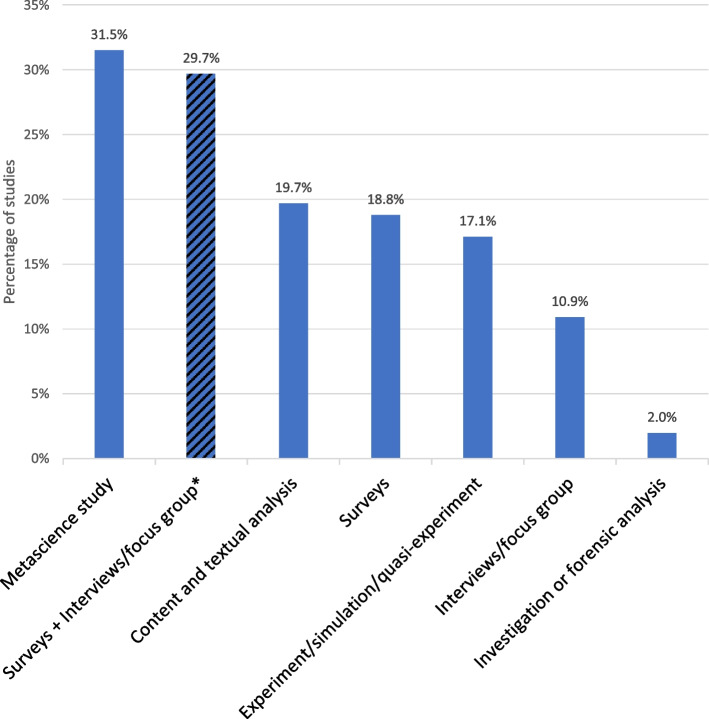
General methodology. *Legend.* This is a category on the methodologies used in the RI empirical literature. *The “Surveys + Interviews/focus group” striped line is the addition of lines present elsewhere in the figure (“Surveys” and “Interviews/focus group”). The total of articles (100%) is 660

- *Q3: What methodologies are prevalent in the empirical literature on RI?* How does one empirically study RI? Are experiments possible? (Fig. 4)

The most favored methodology for studying research integrity is metascience, which accounts for 31.5% of the research. Close behind are quantitative surveys and qualitative interviews/focus groups, making up 29.7% of the studies. Surprisingly, experiment/simulation/quasi-experiment studies come third, representing 17.3% of the total.

**Fig. 5:**
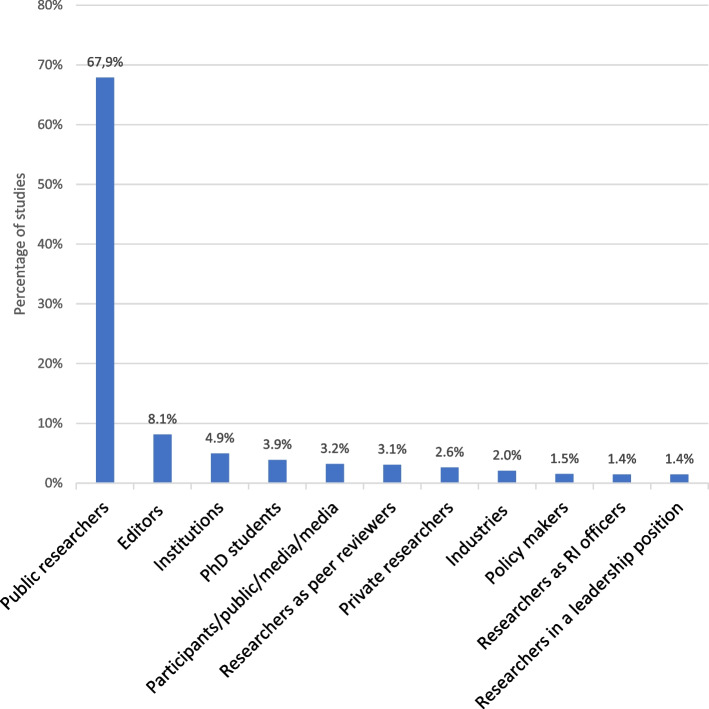
Population or organization studied. *Legend*. Representation of the actors studied in the papers. The total of articles (100%) is 660. RI = research integrity

- *Q4: What populations or organizations are studied in the empirical literature on RI?* How much is it focused on researchers and which kinds? (Fig. 5)

As expected, an overwhelming majority of studies (73.3%) focus on scientists. Notably, the emphasis is primarily on public researchers, who constitute 67.9% of the total. In contrast, scientists from the private sector account only for a small fraction (2.6%). Research on decision-makers is likewise limited, with few studies on policymakers (1.5%), or researchers in leadership positions (1.4%). Similarly, research integrity officers are scarcely studied (1.4%), with most investigations concentrating solely on their responsibilities for teaching research integrity rather than their roles in investigating misconduct.

**Fig. 6:**
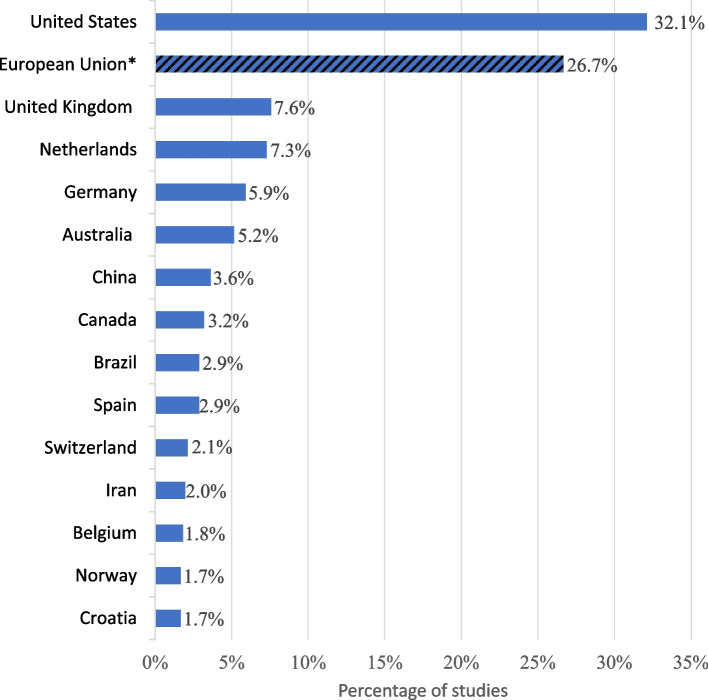
Top 15 of national affiliation of the author.* Legend*. * The “European Union” striped line is the addition of lines present elsewhere in the figure (“Netherlands”, “Germany”, “Spain”, “Belgium” and “Croatia”) and of other countries not included in the figure. The 43 countries not in the top 15 (totaling 133 studies) are not represented but can be found in the Additional file 3. The total of articles (100%) is 660

- *Q5: Where are the empirical studies on RI conducted?* Which countries publish the most research on RI? (Fig. 6)

**Fig. 7:**
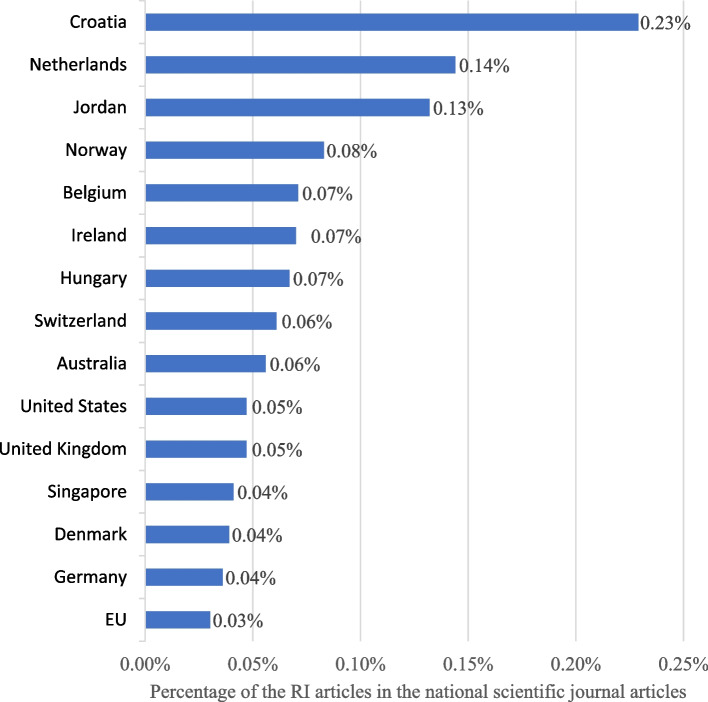
Percentage of the RI articles in the national scientific and technical journal articles.* Legend*. The average of the countries with at least one paper is 0,03%. The 43 countries with less than 4 studies are not represented but can be found in the Additional file 3. The data for the scientific and technical journal articles by countries come from the World Bank Data [17]. The total of articles (100%) is 660. RI = research integrity

The United States is by far the single country with the most publication on RI, representing 32.1% of the total. Northern Europe countries like the United Kingdom, Netherlands and Germany takes the rest of the podium. European Union as a whole comes second with having published 26.7% of articles on RI (Fig. 7).

After normalizing for research output, the United States drops to the 14 th rank and Croatia takes the top spot by a significant margin. The Netherlands, recognized within the European research integrity community for its leadership in institutionalizing RI, takes second place. Jordan is third place and stands out as the only Middle Eastern country above the average.

**Fig. 8:**
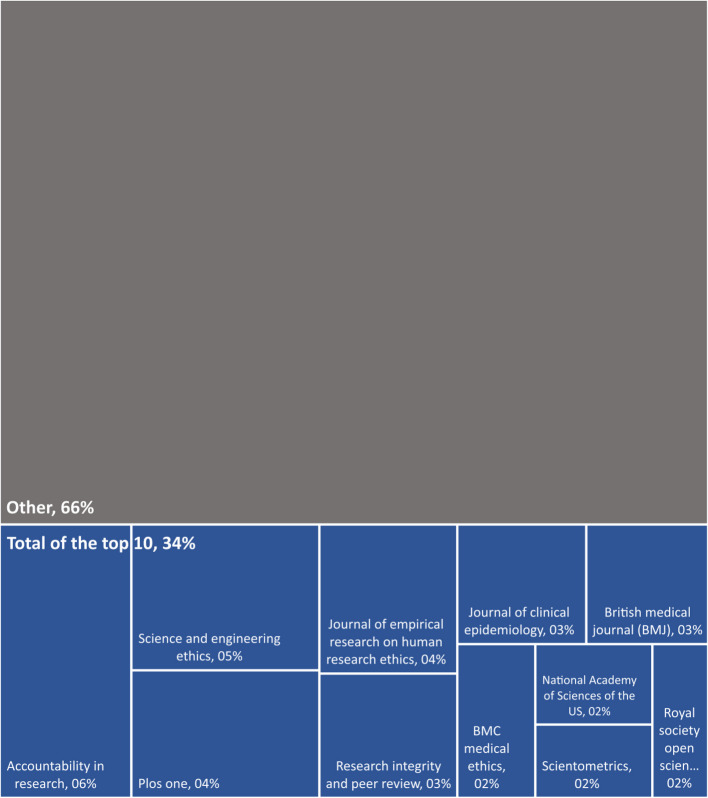
Top 10 journals by the number of empirical publications on RI. *Legend. *There was a total of 333 (50%) unique journal occurrences (i.g 50% of journal who have published on RI only published a single article on RI.) Only the top 10 journal that published the most on RI are named in the figure. The full list of journals can be found in the Additional file 3. The total of articles (100%) is 660

- *Q6: Where is the empirical literature on RI published?* Which journals publish the most empirical research on RI? Which disciplines contribute the most to RI literature? (Fig. 8)

**Fig. 9:**
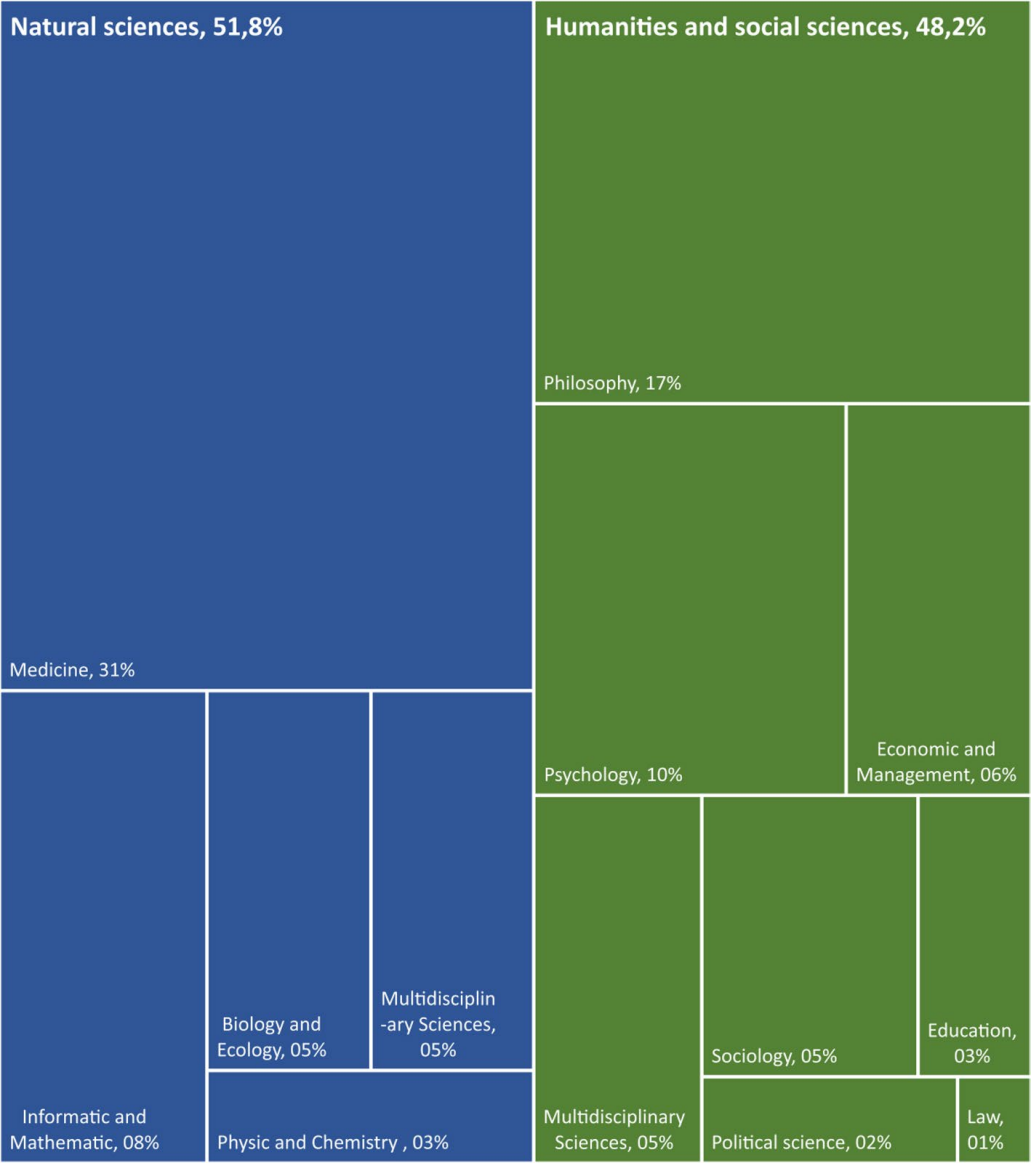
Number of articles per discipline. *Legend.* This figure sums the"Web of Science categories"automatic attribution into broader disciplinary groups. Their exact composition can be found in the Additional file 3. The total of articles (100%) is 660

Most articles (53%) were published in journals that contributed only one article on RI. This is indicative of a highly dispersed field of research. Nevertheless, a clearly identifiable core of specialized journals exists. The top 10 most productive journals account for 33.6% of the total output. Four of the top five journals specialize in RI, while the fifth, Plos one is a mega journal (e.g. a journal with a very broad coverage. See the work of Lăzăroiu for more details on mega journal [18]). As the field continues to grow and establish itself as an independent domain of research, it will be interesting to observe whether the number of unique journals occurrences decrease, reflecting a trend toward more specialization (Fig. 9).

A clear characteristic of the empirical RI corpus is its diversity, encompassing nearly all scientific fields. The natural sciences represent a slight majority, accounting for 51.8%. The three dominant domains are then: medicine (30.8%), philosophy (16.5%), and psychology (10%).

- *Q7: To what degree is the general literature on RI grounded in empirical research? *What is the proportion of empirical versus theoretical research? (Table 1)

**Table 1:** Empirical coverage

Coverage	Percentage
Empirical	33.5%
Theoretical	66.5%

Legend. The decision to apply an empirical keyword filter prevented us from directly calculating the ratio of empirical to theoretical studies. However, by conducting an additional search without the empirical filter keywords (“method”, “result”), we were able to estimate the empirical coverage. Detailed calculations can be found in Additional File 3, and the methodology is further explained in Additional file 4

## Discussion

 By comparing the seven questions covered in the previous section, with the Aubert Bonn and Pinxten [4] study, we can find additional insights about the evolution of the RI field over the past decade. In doing so, we identified six noteworthy trends.

Our findings indicate that empirical studies constitute 33.5% of the total literature on RI.

### A more systematic approach

Two intuitive hypotheses regarding the origin of scientific misconduct are prevalent in the RI literature. One attributes the issue to the occasional"Bad apple"and emphasizes education and training as potential solutions. The other attributes misconduct to the way science is organized, referring to it as a"Wicked system". This perspective favors structural or systemic reforms as their preferred solution. They are frequently referred to in practice, as seen among science journal editors [19] and RI research programs like “Beyond Bad Apples” [20]. In their study, Aubert Bonn & Pinxten [4] highlighted a"Mismatch Between What We Know and What We Propose,"revealing that while the determinant research focused on the “Wicked system” (systemic causes), the proposed solutions were using the “Bad apple” model (targeted individuals). Eight years later, our findings show that this discrepancy has significantly diminished. Most recent studies (52%) advocate for systemic solutions.. This marks an improved alignment between the types of causes investigated and the solutions proposed. The prominence of the “Bad Apple” hypothesis has declined from 54 to 30%, whereas the “Wicked System” hypothesis has risen from 46 to 52%. Moreover, we observed a sharp decline in research focused on"RI training, education, and mentorship,"which dropped from 18% to just 3,8% of the topics studied. This shift can be linked to the reduced focus on the"Bad apples"hypothesis, which traditionally emphasized education, and a growing emphasis on the"Wicked system"hypothesis, which advocates for organizational reforms.

### Increased methodological diversity

Aubert Bonn & Pinxten [4] reported that, surveys, interviews, and focus groups constituted half (51%) of the methodologies used, while in the current study, they account for only 30%. This decrease can be interpreted as an improvement, as surveys on sensitive topics like research misconducts are known to be prone to biases that are difficult to control [21, 22]. The decreased reliance on these methods has allowed for greater diversification in research approaches, particularly benefiting meta-science methodologies. The use of the metascience methodology has increased significantly, rising from the third most employed approach (17%) to the most commonly used method (31.5%). Additionally, we were surprised by the experiment/simulation/quasi-experiment methodologies being now the third most represented (17.3%). This is higher than we anticipated given the inherent challenges associated with conducting experiments in this field. Most of these studies are kinds of modeling of science.

### From description to solution

The focus of RI literature has shifted notably over the past decade. In the Aubert Bonn & Pinxten [4] study, the main focus was on"describing the problem"and"strengthening reporting standards,"which together represented 69% of the research at the time. This earlier emphasis was primarily on understanding the scope of RI issues. However, between 2015 and 2023, the focus has evolved toward solution-oriented research. The two most common themes in our recent period are"Assessing an approach's efficacy"and"Capacity building,"both of which aim to identify or create effective ways to improve RI. These themes now account for 56,3% of the research output. Descriptive studies, which once dominated, have decreased down to 43,7%.^6^ This shift shows how the field has matured as it went from identifying problems to seeking solutions.

### Increased globalization

In the Aubert Bonn & Pinxten [4] study, only six countries had produced ten or more articles on RI. By contrast, in the current landscape, this number has more than doubled, reaching fourteen countries. The United States previously accounted for over half of the literature on RI, has seen its dominance decrease. Although it remains the most productive country, its total share of RI publications has dropped to 32%. This shift suggests that research on RI is becoming more globally distributed, even though North America and Northern Europe continue to lead both in absolute term and when normalized by overall research output. Notably, China ranks only 53rd, significantly below the average. To put this in perspective, relative to its research output, Croatia has published 100 times more papers on RI than China. This slow research output on RI appears to be an Asian phenomenon overall rather than being unique to China. The only exception in the region is Singapore, an early proponent of RI and host of the Second World Conference on Research Integrity [23].

### Empirical coverage of the field

This study found that empirical works account for 33.5% of the total, closely matching the 35% reported by Aubert Bonn & Pinxten. The proportion of empirical work remains relatively low for a field that deals with practical concerns. Aubert Bonn & Pinxten had already noted this discrepancy in their 2019 article and proposed an explanation that may still holds true today:

“Few of the authors of articles on RI are engaged full time in RI, and that collaborators and target audience sometimes spread through an array of distinct disciplines, it may still be challenging to engage in empirical works on the topic.” (pp 12).

### Unresolved and newly identified gaps

We identified three points that could inspire future research in the field of RI. Two of these were already noted in the previous scoping review: the lack of studies on peer review and decision-makers. The last one, a lack of research on the private sector is newly identified.

Given the heavy criticism that peer review has faced (see [24, 25] for examples), along with the rise of various alternatives like registered reports, preprints, and platforms like PubPeer we expected peer review to receive more attention. Despite these factors, there is very little empirical research on peer review in relation to RI. Comparative studies examining the effectiveness of different peer review models would greatly benefit the cause of RI but are notably absent. While it would be methodologically challenging to systematically assess the effectiveness of different peer review approaches or their alternatives, such research would be both innovative and highly valuable for the scientific community.

The lack of studies on decision-makers and the private research sector may be attributed to the difficulty of accessing these groups. Unfortunately, this gap in the literature is increasingly problematic since both groups are becoming more important. Few studies have looked at the organizational or leadership level, despite the key role these individuals play in guiding the research community toward greater integrity, particularly in the context of the growing emphasis on the "Wicked systems" hypothesis. The lack of research on the private sector is likewise concerning. It is roughly three times larger than the public research sector. According to an OECD report [26], in 2020, the public sector accounted for only 25.5%^7^ of research performed in OECD countries, while the private sector made up 74.5%.^8^ The private sector is also not immune to research misconducts. One prominent example is the Industrial Bio-Test Laboratories (IBTL) case, where the company likely falsified 618 clinical trials and toxicity tests in the 70 s [27]. More recently, the highly publicized Theranos case involved faked blood-testing technologies, with irregularities surrounding falsified patents, as discussed by Vincent [28]. On the topic of patents, it is particularly concerning that we found no articles mentioning them, even though they are increasingly seen as equivalent to publications in terms of individual career advancement or institutional prestige.

RI literature should pay greater attention to decision-makers and private researchers. Addressing these gaps is essential for maintaining the relevance of the field in light of evolving research dynamics.

### Study limitation

Our study did not adhere to a formal review protocol, meaning that no preregistration was done. Our approach evolved too rapidly at the beginning of the project. Although we initially planned to replicate the methodology of Aubert Bonn & Pinxten [4], it soon became clear that significant modifications^9^ were necessary to better address our research questions. These adjustments were made throughout the project, which made preregistration incompatible with our exploratory approach. The comparisons done in the discussion section with the Aubert Bonn & Pinxten [4] study should be interpreted with caution. Even if we started with their study as a template, we used different databases, methodologies, and classifications. See additional file 2 for a detailed comparison of our methodologies.

This study may have missed valid publications for four reasons.

1) It is important to acknowledge the issue of articles not being immediately indexed by databases, a phenomenon known as the"rolling wall."Since our study was conducted in early 2024 and includes studies published until late 2023, it's likely that some relevant studies may have not yet been indexed. This issue is particularly pronounced with Constellate, which has a slower rolling wall compared to Web of Science.

2) While including other databases such as Google Scholar, Scopus, and PubMed would have enhanced the comprehensiveness of our study, these were not used due to limited resources and the minimal expected gains, as detailed in the additional file 4.

3) We excluded grey literature (e.g., works that did not passed academic peer review). This is due to the fact that our databases (Web of science and Constellate) are unlikely to have systematically indexed grey literature which open the door to a selection bias. 

4) Finally, we excluded articles that were in preprints or listed in the Predatory Journal List [16], although some of these might be valid. While we did not assess the quality of the included works, we aimed to ensure that only articles meeting minimal peer review standards were considered. 

The process of determining how to categorize each study always involves a degree of subjectivity. This limitation is further compounded by the absence of an independent reviewer, a factor known to reduce bias [29]. While the study would have been stronger with an independent duplicate, the individual categorization of each of the 3,282 articles is organized and openly available in additional file 3. We made it so anyone can freely verify or replicate the categorization process.

Apart from a very basic correspondence check using the Predatory journal list [16] to eliminate articles from predatory journals, we did not assess the quality of the studies. Given that our analysis focuses on research topics and methodologies rather than on study outcomes, the quality of the studies will not affect our findings.

The empirical coverage was approximated rather than directly calculated. See the additional file 3 for the calculations and the additional file 4 for the methodology.

Limiting our search to English-language articles likely introduced a bias, as certain countries publish a significant portion of their research in their native languages. This is especially relevant for China. The Aubert Bonn & Pinxten [4] study faced the same limitation: “China, which is rapidly becoming an important player in scientific publishing worldwide, was scarcely represented in our sample. It is possible that the language limitations of our study (i.e., we only included articles in English) contributed to this disparity” (pp 12).

## Conclusion

Our findings highlight the growing importance of meta-science methodologies, a shift in focus toward problem-solving, and a move away from the"Bad apple"in favor of the"Wicked system"hypothesis.

We also identified three research gaps likely to become increasingly important: research on decision-makers (such as scientists in leadership roles, policymakers, and institutions), the private research sector as well as patents, and the peer review system.

## Supplementary Information

Additional file 1. PRISMA-ScR Checklist. PRISMA good practice checklist for scoping review.

Additional file 2. Correspondence table. The list of motivated modifications to the classification process from the Aubert Bonn & Pinxten methodology.

Additional file 3. Dataset. The complete dataset as well as the various calculations done throughout the project.

Additional file 4. Detailed methodology. Detailed methodology, most notably the search queries and the validity analysis of the comparisons.

## Data Availability

The datasets supporting the conclusions of this article are included within the article and its additional files.

## Abbreviations

RI: Research Integrity
FFP: Fabrication, Falsification, Plagia
QRP: Questionable Research Practice
URP: Unacceptable Research Practice

## Footnotes

1 In hindsight, Constellate only had 8.2% more articles from the humanities and social sciences on research integrity than Web of Science.

2 “responsible innovation”, “responsible research”, “responsible conduct of research” and “metascience”.

3 It is important to note that we still exclude grey literature (e.g., works that did not passed academic peer review). This is due to the fact that both databases are unlikely to have systematically indexed grey literature which open the door to a selection bias.

4 The most recent ALLEA code (14) no longer use the QRP term but URP (unacceptable research practice) instead. We choose to stick with the QRP terminology as the literature has not yet adopted the new term.

5 Although it can be arduous to advocate for a clear-cut distinctions between RI, research ethics, and responsible science, this study requires it so as not to stretch itself beyond its means.

6 For more details about the comparisons see the additional file 2.

7 Percentage of Gross domestic expenditure on R&D (GERD) performed by the higher education. sector 16.2% + performed by the government sector 9.3%

8 Percentage of Gross domestic expenditure on R&D (GERD) performed by the business enterprise. sector 72.1% + performed by the private non-profit sector 2.4%

9 All of the modifications and their motivations can be found in the additional file 2.
