# Supplementary material for: From 2015 to 2023, eight years of empirical research on research integrity: a scoping review
Source: Res Integr Peer Rev. 2025 Apr 30;10:5. doi: 10.1186/s41073-025-00163-1 (PMC12042460; doi:10.1186/s41073-025-00163-1)
Supplement: Supplementary file 4 — Additional file 4. Detailed methodology. Detailed methodology, most notably the search queries and the validity analysis of the comparisons. [file 41073_2025_163_MOESM4_ESM.docx]

**Queries**

All queries were limited to work in English and set to gather from 2016 included to 2023 included.

Main query used in Web of Science: (TS=("responsible innovation")OR TS=("questionable research practices") OR TS=("research misconduct") OR TS=( "research integrity") OR TS=("responsible research") OR TS=(“responsible conduct of research “)OR TS=("scientific misconduct”) OR TS=(“scientific integrity”) OR TS=(“scientific fraud”) OR TS=("metascience")) AND ((ALL=("method") OR ALL=(“methods”) OR ALL=(“result”) OR ALL=("results")))

Main query used in Constellate (JSTOR): ((((((((((“responsible innovation”) OR “questionable research practices”) OR (“research misconduct”)) OR (“research integrity”)) OR ("responsible research")) OR (“responsible conduct of research”)) OR (“scientific misconduct”)) OR (“scientific integrity”)) OR (“scientific fraud”)) OR ("metascience")) AND ((((“method”) OR (“methods”)) OR (“result”)) OR (“results”)).

Additional query used in Web of Science for the broad keywords lexical analysis: (TS=("responsible innovation") OR TS=("responsible research") OR TS=(“responsible conduct of research “)OR TS=("metascience")) AND ((ALL=("method") OR ALL=(“methods”) OR ALL=(“result”) OR ALL=("results")))

Additional query used in Constellate (JSTOR) for the broad keywords lexical analysis: ((((“responsible innovation”) OR ("responsible research")) OR (“responsible conduct of research”)) OR ("metascience")) AND ((((“method”) OR (“methods”)) OR (“result”)) OR (“results”)).

Additional query used in Web of Science for the precise keywords lexical analysis: (TS=("questionable research practices") OR TS=("research misconduct") OR TS=( "research integrity") OR TS=("scientific misconduct”) OR TS=(“scientific integrity”) OR TS=(“scientific fraud”) AND ((ALL=("method") OR ALL=(“methods”) OR ALL=(“result”) OR ALL=("results")))

Additional query used in Constellate (JSTOR) for the precise keywords lexical analysis: ((((((“questionable research practices”) OR (“research misconduct”)) OR (“research integrity”)) OR (“scientific misconduct”)) OR (“scientific integrity”)) OR (“scientific fraud”)) AND ((((“method”) OR (“methods”)) OR (“result”)) OR (“results”)).

Additional query used in Web of Science for the empirical coverage: (TS=("responsible innovation")OR TS=("questionable research practices") OR TS=("research misconduct") OR TS=( "research integrity") OR TS=("responsible research") OR TS=(“responsible conduct of research “)OR TS=("scientific misconduct”) OR TS=(“scientific integrity”) OR TS=(“scientific fraud”) OR TS=("metascience")

Additional query used in Constellate (JSTOR) for the empirical coverage: ((((((((((“responsible innovation”) OR “questionable research practices”) OR (“research misconduct”)) OR (“research integrity”)) OR ("responsible research")) OR (“responsible conduct of research”)) OR (“scientific misconduct”)) OR (“scientific integrity”)) OR (“scientific fraud”)) OR ("metascience"))

**Database selection comparison**Aubert Bonn & Pinxten (1) used three databases: PubMed, Web of Science, and SCOPUS. In this study, we utilized two: Web of Science and Constellate. The difference in database is likely to be a source of bias for the comparison. However, we want to argue in the section that it is mild. The main sub-database of PubMed, MEDLINE, is included in Web of Science (2)^^[[1]](#footnote-1)^^. PubMed also includes two other sub-databases: PubMed Central, which is one-third the size of MEDLINE and serves as an open archive for research funded by the NIH, and Bookshelf, which is smaller still and includes non-peer-reviewed works. Any unique entries from PubMed are likely to be of low quality or irrelevant to our review; therefore, we excluded PubMed to conserve resources. SCOPUS and Web of Science are highly similar. According to Bramer et al. (3 pp10), there is a “large overlap of Scopus and Web of Science,” a finding supported by studies from Martín-Martín et al. (4, 5), which show that 82.4% of citations in 2018 and 83% of citations in 2021 found in SCOPUS are also present in Web of Science. Therefore, we excluded SCOPUS, to save resources as Web of Science sufficiently covered the literature. The inclusion of Constellate did not introduce a significant bias in comparisons with Aubert Bonn & Pinxten, as only 13.7% of the included papers came from this database, and their distribution across study characteristics was broadly similar to those from Web of Science^^[[2]](#footnote-2)^^.

**Inclusion criteria comparison**Our inclusion criteria are the same used in the Aubert Bonn & Pinxten study, with two notable exceptions. They excluded all articles that presented methodological improvement. Although we did not include standard methodological improvements, we did include studies that aim to develop methods for enhancing research integrity within their discipline. Rather than being designed to uncover new facts or increase accuracy, the methodological improvements we include are intended to decrease the vulnerability of the field to falsification or questionable research practices. For instance, the study titled “Controlling Decision Errors with Minimal Costs: The Sequential Probability Ratio t Test” (8) proposes a methodological improvement that makes falsification or spinning more difficult. Although Aubert Bonn & Pinxten likely would not have included this study, we chose to do so because it explicitly introduces a new method to enhances RI.
Furthermore, while Aubert, Bonn, and Pinxten excluded systematic reviews from their analysis, we chose to include them. The secondary data they produce can be collected, manipulated, and analyzed similarly to primary data. Moreover, including these reviews enhances the comprehensiveness of our study.

**Classification process detailed methodology**

Some characteristics can be prone to overlap. For instance, it is not uncommon for articles to survey both researchers and PhD students. In cases where multiple populations, methodologies, or objectives are examined, we opted to assign all relevant characteristics to ensure maximum precision. To prevent studies from being counted more than once in the results, thereby leading to percentages exceeding 100%, studies featuring multiple characteristics were fractioned according to the number of characteristics coded within each category. To illustrate, using the previous example, a study involving both researchers and PhD students would be coded as "researchers + PhD students," resulting in 0.5 counts for researchers and 0.5 for PhD students.
However, an exception to this rule applies to the "Topic of interest" category. Following the aforementioned rule to this category would diminish the value of its analysis. Recognizing that almost all studies on RI are related to QRP or FFP doesn't provide much insight. Therefore, only one characteristic was coded for this category. When it was unclear which one should be attributed, priority was given to the paper's most direct theme. For example, an article about guidelines addressing QRP would be coded as "Guidelines and policies" rather than "QRP + Guidelines and policies."

Topics of interest – What topics does the empirical literature on RI explore?

- Only QRP
- Only FFP
- FFP and QRP
- Guidelines and policies
- Research infrastructures and environments
- Research on RI
- Research integrity training, education, and mentorship
- Publication ethics
- Peer review

This category is designed to be coarse grain and aims to capture what is the object studied by the researchers. It gives a general overview of the themes most addressed in the field.

Aims and sub-aims – What are the primary objectives of the empirical literature on RI? Does the empirical literature on RI primarily focus on identifying individual transgressions or systemic flaws?

- Allegations, sanctions, disclosure of cases
- Level of awareness and compliance
- Exploration of the consequences of misconduct
- Assess an approach efficacy
  - Personal
  - System
  - Mixed
- Capacity building
  - Personal
  - System
  - Mixed
- Research of determinants
  - Personal
  - System
  - Mixed
- Denounce or detect FFP
- Denounce or detect QRP
- Finding the occurrence of only FFP
- Finding the occurrence of only QRP
- Finding the occurrence of FFP and QRP
- Other

This category codes in a finer grain the focus of the research. Its objective was to characterize the general goals of the authors.
The difference between “Denounce or detect” and “Occurrence” can be ambiguous. The former are inquiries on novel or poorly defined FFP/QRP and typically discuss whereas a practice should be considered an FFP or a QRP. The latter measures the abundance of already well-known and relatively well-defined QRP or FFP. We can take the paper “Pitfalls and potentials in simulation studies: Questionable research practices in comparative simulation studies allow for spurious claims of superiority of any method^[[3]](#footnote-3)^” as an example of a “Denounce or detect QRP” paper. The authors are not looking for the prevalence of the QRPs but are only demonstrating their potential for harm and feasibility.

General methodology – What methodologies are prevalent in the empirical literature on RI?

- Experiment/simulation/quasi-experiment
- Meta study
- Content and textual analysis
- Investigation or forensic analysis
- Interviews/focus group
- Surveys

Population or organization studied – What populations or organizations are studied in the empirical literature on RI?

- Researchers as peer reviewers
- Public researchers
- Private researchers
- Researchers in a leadership position
- PhD students
- Researchers as RI officers
- Editors
- Industries
- Institutions
- Participants/public/medias
- Policy makers

The distinction between "Institutions" and "Policy makers" might require further clarification. Articles taking "Institutions" as a subject examine organizations, while "Policy makers" studies look at the individuals at the head of these organizations.
Depending on the methodology used, populations may have been studied indirectly. For instance, examining the occurrence of researcher FFP through retraction notes studies researchers, even though it is done through the editors' outputs.
Since practically all papers published worldwide are authored by public researchers, we have assumed that when an article examines the scientific literature, its subject is "Public researchers".

National affiliation of the author – Where are the empirical studies on RI conducted?
Only the national affiliations of the first author were used. In cases of multiple affiliations, they were coded and fractioned into their respective nationalities. We represented only the top 15 most productive countries. The complete list can be found in the Additional file 3.

Percentage of the RI articles in the research output – Where are the empirical studies on RI conducted?
An additional analysis can be done to find which country produced the most research on RI relative to its overall research output. The World Bank data provided the research output data. The dataset can be found on their website (9). Countries with less than four articles were not represented in the result section. Taiwan was left out, as the World Bank Data doesn’t collect its data. The complete list can be found in the Additional file 3.

Number of articles per discipline – Where is the empirical literature on RI published?
The most straightforward way to answer the question is to look for which journals have the most publications on RI. We used the standard metadata provided by the databases to count the number of publications per journal.

Discipline affiliation of the study by the journal – Where is the empirical literature on RI published?
To answer the thematic question we looked the scientific disciplines affiliation of the journals. We utilized the "Web of Science categories" automatic setting to fulfill this category. A few corrections have been made when the automated system gave different discipline affiliations to the same journal. We manually coded the affiliations for Constellate studies using the Web of Science categories as a template. The discipline categories provided by Web of Science are rather fine-grained. To still have a large-scale view, we summed the Web of Science categories into broader ones. The exact compositions can be found in the Additional file 3.

**Additional searches**

Empirical coverage – To what degree is the general literature on RI grounded in empirical research?

The use of an empirical keyword filter prevents us from directly calculating the percentage of empirical versus theoretical studies, as we don’t know the total number of theoretical studies on RI. However, we can estimate this number with an additional search. We already know how many theoretical studies passed through the empirical keyword filter, but we are interested in the number that the filter blocked. To estimate this, we performed a search without the empirical filter to retrieve all articles related to RI. By subtracting the number of studies that passed the filter from the total number retrieved, we can estimate how many theoretical studies were blocked. Next, to isolate the theoretical studies, we need to remove other types of studies. We do this by adjusting for the proportion of studies excluded due to criteria other than being theoretical (such as being off-topic or incomplete).

Our estimation relies on two key assumptions. First, we assume that the proportion of studies excluded by non-theoretical criteria remains consistent, regardless of the filter. Second, we assume the empirical keyword filter did not inadvertently block any empirical studies. While these assumptions may not hold perfectly, we believe they are reasonable enough to provide a reliable approximation. Finally, we combine the estimated number of theoretical studies blocked by the filter with the known number of theoretical studies that passed through it. This gives us an approximate total for theoretical studies on RI, allowing us to calculate the estimated ratio of empirical to theoretical studies.

**Bias estimation of the comparisons**

Two key factors must be considered when assessing validity:
Are the characteristics being compared the same?
If categories have been added or eliminated from Aubert Bonn and Pinxten, can they be redistributed according to our criteria?

Below is a list of the comparisons we made with Aubert Bonn and Pinxten in our paper:

**Characteristic: Methodology Used**
Overall, this characteristic is only minimally biased. The only category removed was “Tool building or validation,” which accounted for only 6 of the 342 studies in the Aubert Bonn and Pinxten study. Redistributing these six studies among the other categories would introduce a maximum positive bias of 0.3% (if all were allocated to one category), which is negligible.

Categories: Interviews and Surveys
“The use of interviews and surveys decreasing from 51% to 30%.”
We subdivided the interviews and surveys categories, whereas Aubert Bonn and Pinxten did not differentiate between the two. However, as long as we combine interviews and surveys for the comparison which we take care to do, the categories are equivalent.

Categories: Meta-scientific Methods
“Meta-scientific methods increased from 17% to 31.5%.”
The only change we made was to include systematic reviews in this category. Aubert Bonn and Pinxten identified three systematic reviews. Adding these to their meta-scientific methods category raises the percentage slightly from 17% to 17.8%, which is insufficient to compromise the comparison.

**Characteristic: Sub-aim**

Category: systematic factors

““Wicked system” rose from 46% to 52%”

Category: personal factors
““Bad Apple” intuitive hypothesis declined from 54% to 30%”

These comparisons could be subject to significant bias, but we have already accounted for this. In our methodology, we removed the “awareness and compliance” characteristic. However, Aubert Bonn and Pinxten provide sufficient information in their Figure 5 to allow us to redistribute the “awareness and compliance” studies between systematic and personal factors according to our methodology. The resulting values (46% and 54%) reflect what we would expect if our methodology were applied to their data. (See Additional File 2 for details on how the “awareness and compliance” characteristic was reassigned.)

**Characteristic: Ad-Hoc**

Category: Testing Solutions
“testing solutions (rising from 31% to 56%)”

Category: Describing the Problem
“describing the problem (declining from 69% to 44%)”

For this comparison, we did not modify Aubert Bonn and Pinxten’s methodology. We classified the studies in our dataset based on their criteria. Because these criteria were topic-based, we used our “topic” category for the classification rather than individual studies. The list of topics categorized as either descriptive or solution-testing (according to Aubert Bonn and Pinxten’s criteria) is available in Additional File 2.

**Characteristic: Topic**

Category: RI Training, Education, and Mentorship
“sharp decline in research focused on "RI training, education, and mentorship," which dropped from 18% to just 3,8% of the topics studied”

These characteristics were not modified except for a superficial name change for “RI training, education, and mentorship.” However, three categories—REC/IRB, cheating and academic misconduct and allegations, and sanctions/disclosures of cases—were removed in our methodology. In the Aubert Bonn and Pinxten study, these removed categories account for 33 studies that then need to be redistributed among the remaining categories. In the worst-case scenario, if all 33 studies were reallocated to one category, this would result in a maximum positive bias of 10% (i.e., increasing from 18% to 28% for “RI Training, Education, and Mentorship”). Because this bias is only positive, it does not invalidate the first comparison.

All nationality comparisons remain unaffected, as we applied the same methodology uniformly.

**References:**
1. Aubert Bonn N, Pinxten W. A decade of empirical research on research integrity: what have we (not) looked at? J Empir Res Hum Res Ethics. 2019;14(4):338-352. <https://doi.org/10.1177/1556264619858534>

2. Clarivate. MEDLINE on Web of Science. Clarivate. 2023 Apr 23. <https://clarivate.com/products/scientific-and-academic-research/research-discovery-and-workflow-solutions/webofscience-medline/>. Accessed Mar 2024.

3. Bramer WM, Rethlefsen ML, Kleijnen J, Franco OH. Optimal database combinations for literature searches in systematic reviews: A prospective exploratory study. Syst Rev. 2017;6(1):245. <https://doi.org/10.1186/s13643-017-0644-y>

4. Martín-Martín A, Orduna-Malea E, Thelwall M, Delgado López-Cózar E. Google Scholar, Web of Science, and Scopus: A systematic comparison of citations in 252 subject categories. J Informetr. 2018;12(4):1160‑1177. <https://doi.org/10.1016/j.joi.2018.09.002>

5. Martín‐Martín A, Orduña‐Malea E, López‐Cózar ED. Coverage of highly-cited documents in Google Scholar, Web of Science, and Scopus: a multidisciplinary comparison. Scientometrics. 2018;116(3):2175–2188. <https://doi.org/10.1007/s11192-018-2820-9>

6. Martín-Martín A, Thelwall M, Orduna-Malea E, Delgado López-Cózar E. Google Scholar, Microsoft Academic, Scopus, Dimensions, Web of Science, and OpenCitations’ COCI: A multidisciplinary comparison of coverage via citations. Scientometrics. 2021;126(1):871‑906. <https://doi.org/10.1007/s11192-020-03690-4>

7. Chavarro D, Ràfols I, Tang P. To what extent is inclusion in the Web of Science an indicator of journal ‘quality’? Res Eval. 2018;27(2):106‑118. <https://doi.org/10.1093/reseval/rvy001>

8. Schnuerch M, Erdfelder E. Controlling decision errors with minimal costs: The sequential probability ratio t test. Psychol Methods. 2020;25(2):206–226.

9. World Bank Open Data. Scientific and technical journal articles. World Bank; 2020. https://data.worldbank.org/indicator/IP.JRN.ARTC.SC Accessed Jan 2024.

1. This was not the case when Aubert Bonn & Pinxten started their scoping review in 2005. [↑](#footnote-ref-1)
2. The details of the impact the inclusion of Constellate had on the data coming from Web of Science can be found in the Additional file 3. [↑](#footnote-ref-2)
3. Pawel, S., Kook, L., & Reeve, K. Pitfalls and potentials in simulation studies: Questionable research practices in comparative simulation studies allow for spurious claims of superiority of any method. Biometrical Journal, 2024: 66(1), 2200091. <https://doi.org/10.1002/bimj.202200091> [↑](#footnote-ref-3)
